# Therapeutic Value of Single Nucleotide Polymorphisms on the Efficacy of New Therapies in Patients with Multiple Sclerosis

**DOI:** 10.3390/jpm11050335

**Published:** 2021-04-23

**Authors:** María José Zarzuelo Romero, Cristina Pérez Ramírez, María Isabel Carrasco Campos, Almudena Sánchez Martín, Miguel Ángel Calleja Hernández, María Carmen Ramírez Tortosa, Alberto Jiménez Morales

**Affiliations:** 1 Department of Pharmacy and Pharmaceutical Technology, Faculty of Pharmacy, University of Granada, 18071 Granada, Spain; mjzarzuelo@gmail.com; 2 Pharmacogenetics Unit, Pharmacy Service, Virgen Macarena University Hospital, 41009 Seville, Spain; mangel.calleja.sspa@juntadeandalucia.es; 3 Pharmacogenetics Unit, Pharmacy Service, Virgen de las Nieves University Hospital, 18014 Granada, Spain; mariaisabelcarrascocampos@gmail.com (M.I.C.C.); almuweb06@gmail.com (A.S.M.); alberto.jimenez.morales.sspa@juntadeandalucia.es (A.J.M.); 4 Department of Biochemistry, Faculty of Pharmacy, University of Granada, 18071 Granada, Spain; mramirez@ugr.es

**Keywords:** multiple sclerosis, natalizumab, fingolimod, teriflunomide, dimethyl fumarate, alemtuzumab, cladribine, siponimod, ocrelizumab, response, polymorphisms

## Abstract

The introduction of new therapies for the treatment of multiple sclerosis (MS) is a very recent phenomenon and little is known of their mechanism of action. Moreover, the response is subject to interindividual variability and may be affected by genetic factors, such as polymorphisms in the genes implicated in the pathologic environment, pharmacodynamics, and metabolism of the disease or in the mechanism of action of the medications, influencing the effectiveness of these therapies. This review evaluates the impact of pharmacogenetics on the response to treatment with new therapies in patients diagnosed with MS. The results suggest that polymorphisms detected in the *GSTP1*, *ITGA4*, *NQO1*, *AKT1*, and *GP6* genes, for treatment with natalizumab, *ZMIZ1*, for fingolimod and dimethyl fumarate, *ADA*, for cladribine, and *NOX3*, for dimethyl fumarate, may be used in the future as predictive markers of treatment response to new therapies in MS patients. However, there are few existing studies and their samples are small, making it difficult to generalize the role of these genes in treatment with new therapies. Studies with larger sample sizes and longer follow-up are therefore needed to confirm the results of these studies.

## 1. Introduction

Multiple sclerosis (MS) is a chronic inflammatory demyelinating disease of autoimmune origin, heterogeneous in its etiology and clinical course, in which inflammation and demyelination lead to neurodegeneration and progressive disability [1]. There are 2.5 million cases worldwide, with a mean prevalence of 80–100 cases per 100,000 population per year, varying by country, and 1 per 1000 in the Caucasian population [2]. The female-to-male ratio in incidence of MS is 2 to 1, and it is the leading cause of non-traumatic neurological damage in young adults (aged 20–40) in Europe [2]. There are risk factors for developing the disease, including climate (it is more common in cold than in warm regions), ultraviolet radiation, and vitamin D levels, as well as smoking. In addition, genetics is believed to play an important role in its occurrence [3,4].

This chronic disease is characterized by variable clinical development, making it difficult to diagnose. Clinically, it presents with multiple signs of neurological dysfunction, followed either by recovery or by increased disability due to irreversible functional damage [5]. The destruction of the myelin surrounding the neurons is what causes the lesions and consequent disability characteristic of the disease, which can include vision disorders, difficulty in speaking, tremor, spasms and/or tingling in the extremities, dizziness, fatigue, intestinal and urinary problems, and difficulty in moving the limbs [6,7]. 

Four types of MS can be distinguished, depending on the progression of the disease. The most common is relapsing-remitting multiple sclerosis (RRMS), which presents in the form of attacks that can last from a few hours to months: in other words, recurrent acute or subacute episodes of neurological disturbances followed by recovery with or without some residual deficit [8]. In primary progressive multiple sclerosis (PPMS), the symptoms appear gradually and irreversibly. Secondary progressive multiple sclerosis (SPMS) refers to a deterioration that RRMS patients may experience after some years. Finally, the least common type, progressive relapsing multiple sclerosis (PRMS), is similar to RRMS, but in this case the attacks always get worse [9]. 

Although there is no existing cure for MS, there are treatments that slow the course of the disease and mitigate its symptoms. These can be divided into classical therapies and new therapies. The former are those that have been used for longer and whose action is well known; they involve the use of beta interferon (Avonex^®^, Betaferon^®^, Extavia^®^, Rebif^®^) and glatiramer acetate (Copaxone^®^) [10]. 

The new therapies are based on compounds that have come into use recently and whose characteristics and mechanism of action are largely unknown. They needed to be developed, because in many cases patients do not respond to the classical treatments. Dimethyl fumarate (Tecfidera^®^) and teriflunomide (Aubagio^®^) are used as first-line treatments and natalizumab (Tysabri^®^), fingolimod (Gilenya^®^), alemtuzumab (Lemtrada^®^), cladribine (Mavenclad^®^), siponimod (Mayzent^®^), and ocrelizumab (Ocrevus^®^) as second-line treatments [11,12,13].

These medications show a high rate of response and are capable of reducing the annual attack rate by 31–69% and the progression of the disease by up to 66% [14,15,16,17,18,19,20,21,22,23,24,25]. However, there is a percentage of patients who do not respond to treatment with these drugs. This interindividual variability may be due to genetic alterations. It is known that there are regions of the genome that are frequently subject to genetic variability. Such sites are called SNPs (single-nucleotide polymorphisms) and their diversity gives rise to changes in gene expression or protein activity, among other effects [26]. 

The implementation of pharmacogenomics in clinical practice has great potential to allow more personalized treatment. However, despite the large number of treatment options available to patients with MS and a high degree of variability in the response to these treatments, there is still no reliable pharmacogenomic biomarker that differentiates between those who respond to MS treatment and those who do not. Since MS requires chronic treatment, an early decision on appropriate therapy can be of great clinical benefit to MS patients, slowing disease progression, preventing potential adverse events, and improving treatment efficacy.

The following review assesses the impact of pharmacogenetic studies on response to treatment with new therapies in patients diagnosed with MS. 

## 2. Pharmacogenetics of New Therapies in MS

Genetic alterations involved in the pathological environment of the disease, metabolisms or mechanism of action may modify the effectiveness of new therapies in MS. This review will focus on dimethyl fumarate, teriflunomide, natalizumab, fingolimod, alemtuzumab, cladribine, siponimod, and ocrelizumab (Table 1).

### 2.1. Dimethyl Fumarate

**Figure 1:**
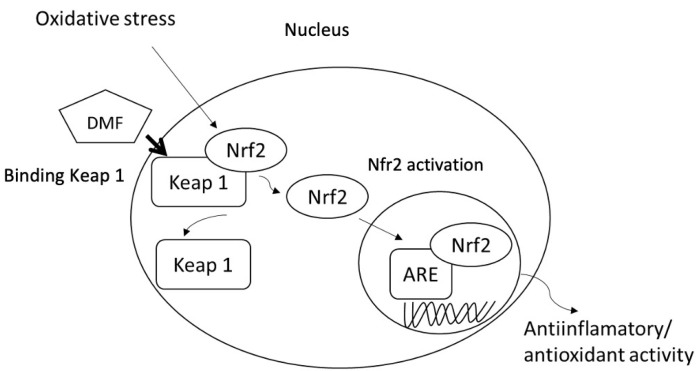
Mechanism of action of dimethyl fumarate.

Dimethyl fumarate (DMF) (Tecfidera^®^) is a fumaric acid ester which activates the nuclear factor erythroid-2-related factor-2 (Nrf2) transcription pathway [27]. Although its mechanism of action is not fully known, this pathway represents a cell defense system against potentially toxic stimuli, including inflammatory and oxidative stress, both of which are presumably involved in the pathogeny of MS; this neuroprotective effect could be due to inhibition of the binding of the Nfr2 transcription factor to its regulatory protein (Keap1). This would allow the transcription factor to accumulate in the cell nucleus and induce the expression of genes related to so-called AREs (antioxidant response elements), involved in eliminating cell toxins, normalizing energy metabolism, and repairing damaged proteins. By activating this pathway, DMF is thought to be capable of reducing the response of inflammatory cells, both peripherally and centrally, and of exerting a cytoprotective effect on the CNS against toxic stimuli, providing a beneficial effect on the pathogenic mechanisms of MS (Figure 1) [28]. Dimethyl fumarate has proved efficacious in patients with RRMS, with a 49% reduction in risk of attacks in a two-year period, as well as a reduction of 53% in the annual relapse rate and of 38% in disease progression [14,15]. Genetic alterations in the following genes have been shown to have a significant influence on DMF treatment [29].

#### 2.1.1. Zinc Finger MIZ-Type Containing 1; ZMIZ1

As with fingolimod treatment, the *ZMIZ1* gene also seems to play a crucial role in treatment with dimethyl fumarate [30]. The influence of SNPs in the *ZMIZ1* gene on response to DMF in patients with MS has not so far been evaluated. However, a study with 39 Caucasian subjects from a cross-section of patients with MS receiving treatment with DMF in Australia and the United States and 40 healthy subjects showed greater expression of the *ZMIZ1* gene in patients treated with DMF compared to those not treated with this drug (*p* = 0.031, CV = 32%) [31]. The effect of the rs1782645 (C>T) polymorphism of *ZMIZ1* on the expression of this gene was also evaluated; the association was not statistically significant [31].

#### 2.1.2. NADPH Oxidase 3; NOX3

The *NOX3* gene is located on chromosome 6q25.1 [32]. It encodes an enzyme found in many types of cells, primarily in the plasma membrane of the inner ear, lung, and cerebral cortex, and catalyzes the production of superoxide by a one-electron reduction of oxygen, using NADPH as the electron donor [33]. The ROSs generated by NOX affect the differentiation of oligodendrocyte precursor cells, as these are highly sensitive to oxidative stress. The development of demyelination in MS lesions is due to loss of oligodendrocytes. It has been suggested that the production of NOX3 induces oligodendrocyte differentiation in patients with MS [34].

A study of 564 Caucasian patients with RRMS in Sweden with indications for starting DMF observed an association of the G allele of the rs6919626 (G>A,T) polymorphism with reduced ROS generation in monocytes after ex vivo stimulation with *E. coli* (b = −0.28; *p* = 0.057) [35]. The same allele was similarly associated with low response to DMF (OR = 1.57; *p* = 0.036) [35].

### 2.2. Teriflunomide

**Figure 2:**
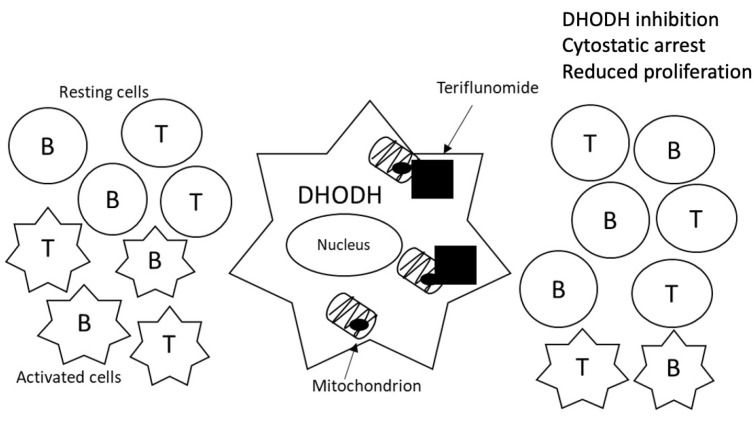
Mechanism of action of teriflunomide.

Teriflunomide (Aubagio^®^) is a specific non-competitive reversible inhibitor of the mitochondrial enzyme dihydroorotate dehydrogenase (DHODH), implicated in the de novo synthesis of pyrimidines in proliferating cells [36,37,38,39]. Thus, teriflunomide blocks the cell cycle in the S (synthesis) phase and exerts a cytostatic effect on proliferation of T and B cells, limiting their participation in the inflammatory processes involved in the pathogenesis of MS (Figure 2) [40]. Teriflunomide has been shown to be efficacious for oral treatment of RMSS [29,41]. It has demonstrated its efficacy by reducing the annual relapse rate by 36% (*p* < 0.01) and disability progression by 27% (*p* = 0.04) [29]. The interindividual variability of response may be influenced by genetic alterations in genes implicated in the mechanism of action of this drug or in the pathologic environment of the disease.

#### 2.2.1. ATP-Binding Cassette, Subfamily G, Member 2; ABCG2

The *ABCG2* gene is located in the 4q22.1 region [42]. It encodes the ABC membrane transporter (ATP-binding cassette), which is responsible for transporting molecules through membranes [42]. 

The only finding that has been described is a relationship with the rs2231142 (C>A) polymorphism in 24 healthy Asian subjects in Korea after administration of the drug, where carriers of the C allele showed a reduction in transporter activity of ABCG2 through an increase in the active metabolite levels of the drug (95% CI for difference between means = −1.94, −0.41; *p* = 0.004) [43].

#### 2.2.2. Dihydroorotate Dehydrogenase; DHODH

The *DHODH* gene is located in region 16q22.2 [44]. It codes for a mitochondrial enzyme which catalyzes de novo pyrimidine biosynthesis in T and B lymphocytes. Inhibition of de novo pyrimidine synthesis is crucial for rapid expansion of lymphocytes, and therefore inhibition of *DHODH* is a strategy in the treatment of autoimmune pathologies such as rheumatoid arthritis and MS [16,45].

The role of this gene in the effectiveness of teriflunomide treatment in MS has not been evaluated. However, in another autoimmune disease, rheumatoid arthritis, it was observed that in 147 Caucasian patients in Poland treated with teriflunomide the rs3213422 (C>A) polymorphism was associated with treatment response. Specifically, patients carrying the C allele showed greater frequency of remission (OR = 1.98; 95%CI = 1.00–3.94; *p* = 0.048) [46].

### 2.3. Natalizumab

**Figure 3:**
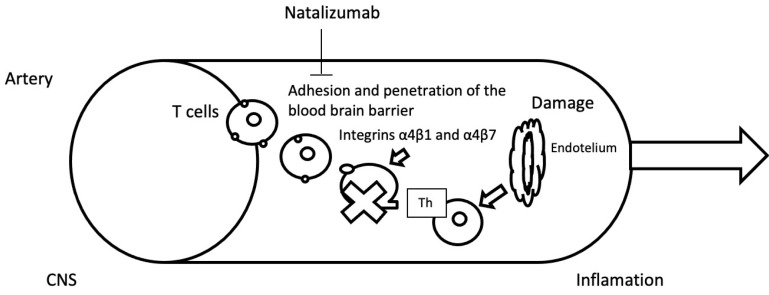
Mechanism of action of natalizumab.

Natalizumab (Tysabri^®^) is a humanized monoclonal IgG4 antibody that binds to the α4 subunit of the α4β1 (VLA-4: very late antigen-4) and α4β7 integrins. It is approved for the treatment of MS and Crohn’s disease [47]. These integrins are responsible for facilitating the passage of pro-inflammatory cells through tissues to reach sites of inflammation [48]; therefore, if this pathway is inhibited, migration of lymphocytes through the blood-brain barrier will be diminished, thereby reducing inflammation in the brain (Figure 3), which is one of the main causes of demyelinating lesions [48]. Additionally, a possible supplementary mechanism of action has recently been discovered, whereby natalizumab affects the regulation of genes involved in the activation of B lymphocytes and neutrophils, reducing the inflammatory process, as well as possible beneficial effects related to the mechanism of oxidative damage reduction in MS patients [49].

Natalizumab is a safe and efficacious drug [50]. It is effective in 75% of cases for the treatment of RRMS in patients that have not responded to first-line treatments such as beta interferon or glatiramer acetate and it reduces the annual attack rate by 68% [17]. It has also proved efficacious in cases of PPMS, since a reduction in relapses of more than 50% has been seen in patients after a year of treatment [47,49].

In spite of these good results, there is individual variability in the therapeutic response to natalizumab. Genetic alterations in genes involved in the mechanism of action of the drug or in the pathologic environment of the disease may play a crucial role in this phenomenon. 

#### 2.3.1. Glutathione S-Transferase Pi 1; GSTP1

The *GSTP1* gene is located on chromosome 11, in region q13.2 [49,51]. It encodes the glutathione S-transferase P protein, an antioxidant enzyme which acts by catalyzing the conjugation of endogenous glutathione [52]. It is believed to influence the progress of MS, as the advance of the disease may be linked to oxidative stress in the body caused by inflammation. 

One of the most extensively studied polymorphisms in the variability of response to natalizumab is rs1695 (A>G, Ile105Val) [49,51]. A study conducted in Greece in 129 Caucasian patients with MS analyzed the possible involvement of the rs1695 polymorphism in the response to natalizumab, showing an improvement in the disability of patients carrying the A allele (χ^2^ = 0.031; df = 1; *p* = 0.861) [49].

#### 2.3.2. Integrin Subunit Alpha 4; ITGA4

The *ITGA4* gene is located in region 2q31.3 [53]. This gene codes for the integrin alpha 4 subunit of the VLA-4 (very late antigen-4) protein, which belongs to adhesion molecules activated during the inflammatory process, facilitating the migration of lymphocytes and monocytes into the central nervous system (CNS) through VCAM-1 (vascular cell adhesion molecule-1) [54,55]. Natalizumab interferes in the interaction between *ITGA4* and VCAM-1, preventing immune cells from migrating to the CNS [17].

We have found no studies evaluating the association of *ITGA4* polymorphisms with natalizumab response; however, a study of 117 Caucasian patients in Slovakia found an association between the AG genotype of the rs1143676 polymorphism (G>A, Gln878Arg) and susceptibility to MS (OR = 1.73; 95% CI = 1.07–2.81; *p* = 0.024) [56].

#### 2.3.3. NAD(P)H Quinone Dehydrogenase 1; NQO1

The *NQO1* gene is located on chromosome 16, region q22.1 [49]. The protein encoded by this gene is an antioxidant enzyme called NAD(P)H quinone oxidoreductase. It acts by catalyzing the two-electron reduction of quinones, preventing their participation in the redox cycle and the subsequent generation of reactive oxygen species (ROSs) [57]. Oxidative stress seems to be involved in the pathogenesis of MS, where ROSs are produced during the inflammatory stage, with the consequent apoptosis of oligodendrocytes [58]. 

In a study of 124 Caucasian patients in Greece, an association of the rs1800566 polymorphism (C>T, Pro187Ser) with response to natalizumab was observed; it was greater in patients with the C allele (χ^2^ = 3.320; df = 1; *p* = 0.068) [49].

#### 2.3.4. AKT Serine/Threonine Kinase 1; AKT1

The *AKT1* gene, located in region 14q32.33, encodes a serine/threonine protein kinase involved in cell survival, proliferation, and growth [59]. This enzyme intervenes in apoptotic processes, and genetic alterations in the gene can give rise to lymphocyte proliferation, modifying the normal process of their apoptosis [59]. Its relationship to MS is based on a reduction in permeability of the blood-brain barrier to the passage of lymphocytes, leading to an increase of these lymphocytes in peripheral blood [59].

A study conducted of 67 Caucasian patients with RRMS in Italy who were fully responsive to natalizumab analyzed the influence of the rs2498804 (G>T) polymorphism on the effectiveness of natalizumab, and observed that carriers of the T allele were associated with a lower lymphocyte count and a lower risk of relapse after completing the treatment (OR = 11.79; 95% CI: 3.72–37.38; *p* < 0.01) [60].

#### 2.3.5. Glycoprotein VI Platelet; GP6

The *GP6* gene is located on chromosome 19q13.42 [61]. The protein it encodes is a collagen receptor which plays a crucial role in collagen-induced platelet aggregation and thrombus formation [62]. The platelet response that occurs in the presence of vascular damage may be reduced due to *GP6*, resulting in poor homeostasis, which causes an accumulation of vascular lesions and a reduction in the cerebrovascular reactivity that causes neurodegeneration in MS [63]. A study of 67 Asian patients with MS in Kuwait found that the C allele for the rs2304166 (C>G) polymorphism was associated with poor response to natalizumab (OR = 22.18; 95% CI: 5.76–95.88; *p* < 0.01) [64].

### 2.4. Fingolimod

**Figure 4:**
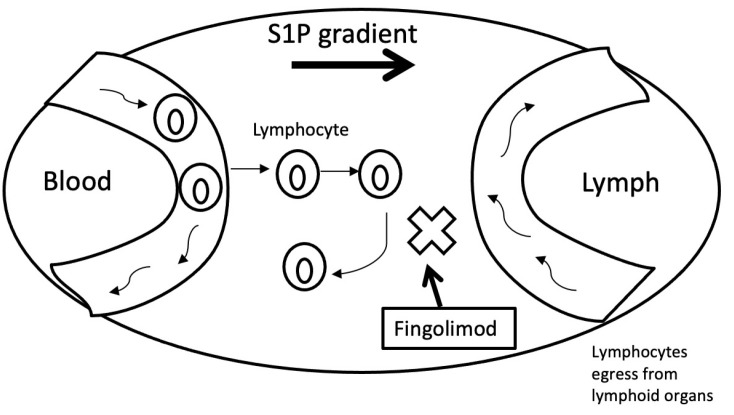
Mechanism of action of fingolimod.

Fingolimod (Gilenya^®^) is a sphingosine-1-phosphate (S1P) analogue and its function in the immunopathology of MS is reversible and selective retention of activated T lymphocytes within the lymph nodes, preventing their migration to the CNS, and therefore the inflammation and demyelination typical of MS. Fingolimod, being an S1P analogue, is phosphorylated in the same way as sphingosine; the product of this enzymatic reaction is fingolimod-P, a drug that binds, with different nanomolar affinity, to the five S1P receptors (S1P1, S1P2, S1P3, S1P4, and S1P5), which are intracellularly coupled to G proteins [65,66]. When it binds to any of these receptors, fingolimod temporarily activates the associated G protein and subsequently internalizes the receptor, blocking the egress of T lymphocytes to the lymph (Figure 4) and thereby preventing their action and damage to CNS cells [67,68].

Fingolimod was the first orally administered drug approved by the FDA for the treatment of RRMS [68]. In numerous multicenter randomized clinical trials, it has been shown to have a good efficacy and safety profile compared to placebo and to beta interferon [69,70].

In a study with 358 Caucasian patients conducted in Italy, fingolimod proved capable of drastically reducing the annual relapse rate in patients without previous treatment (*p* < 0.01) [69]. It has also shown a loss of disease activity in 53% of previously untreated patients and in 36% of patients previously treated with natalizumab (*p* < 0.01). Moreover, it has demonstrated a reduction of 52% in the annual attack rate [18,22]. Despite these encouraging results, there is individual variability in the therapeutic response to fingolimod [69]. Genetic alterations in genes involved in the drug’s mechanism of action may play a crucial role in this phenomenon. 

#### Zinc Finger MIZ-Type Containing 1; ZMIZ1

The *ZMIZ1* gene is located on chromosome 10q22.3 [30]. It is a member of the PIAS family of proteins (protein inhibitors of activated STAT [signal transducer and activator of transcription]), which interact with nuclear hormone receptors, and is a coactivator of transcription factors such as Notch1 (Notch receptor 1) [71]. Notch1 gives rise to oligodendrocyte maturation through myelin proteins and promotes remyelination in degenerative diseases such as MS [72]. 

The influence of polymorphisms on the effectiveness of fingolimod in MS patients has not so far been evaluated. However, a study in Australia and the United States with 39 Caucasian patients and 40 healthy controls showed greater expression of the *ZMIZ1* gene in patients treated with fingolimod compared to those not treated with this drug (*n* = 40) (*p* = 0.039; Coefficient of Variation (CV) = 50%) [31]. It also evaluated the effect of the rs1782645 (C>T) polymorphism of *ZMIZ1* on the expression of this gene; the association was not statistically significant [31].

### 2.5. Alemtuzumab

**Figure 5:**
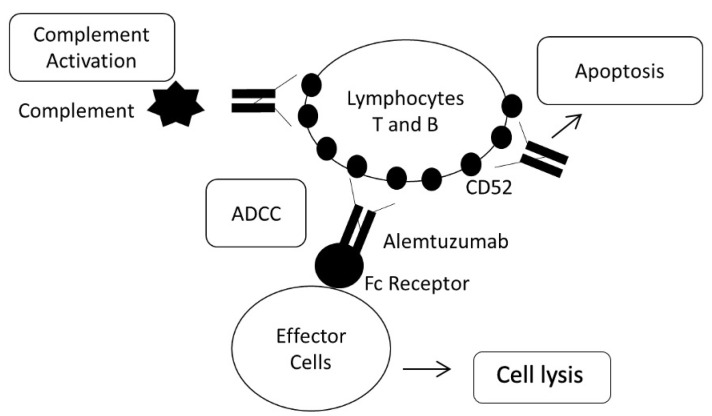
Mechanism of action of alemtuzumab.

Alemtuzumab (Lemtrada^®^) is a humanized monoclonal IgG1 antibody which binds to the CD52 protein. It induces antibody-dependent cellular cytotoxicity (ADCC) and lysis mediated by complement-dependent cytotoxicity (CDC), after binding to the cell surface of T and B lymphocytes. CD52 is expressed on the surface of immune system cells, such as T and B lymphocytes, and as a consequence of its mechanism of action it causes depletion of those cells [73,74,75]. Moreover, alemtuzumab leads to caspase-dependent and -independent apoptosis [76,77,78]. It seems that this reduction of circulating T and B cells and their subsequent repopulation could reduce the possibility of attacks and ultimately slow the course of the disease [79,80]. Alemtuzumab is one of the most effective drugs against RRMS (Figure 5) [81]. It reduces the risk of relapses by 66% and disease progression by 69% [20,21]. In spite of these results there is interindividual variability in the response to the drug, and genetic alterations in genes implicated in its mechanism of action may play an important role in this phenomenon. 

#### 2.5.1. Fc Fragment of IgG Receptors; FCGR2A and FCGR3A 

*FCGR2A* (IIa) and *FCGR3a* (IIIa) are located in the 1q23.3 region [82]. They are receptors of the Fc fragment of IgG and play an important role in protecting the body against antigens [83]. These receptors are present in monocytes, macrophages, neutrophils, natural killer cells, and B and T lymphocytes, and they play a part in modulating the production of antibodies through B cells [83]. Antibody-dependent cellular cytotoxicity requires activation of FcγR, expressed in immune cells. Lymphoid depletion of antibodies such as alemtuzumab leads to lysis of T and B cells, when the Fc fragment of alemtuzumab binds to FcγR, the lymphocytes being less accessible and remaining in the lymphoid tissue and the CNS [80]. A study of 85 Caucasian patients with RRMS in Germany observed no significant association of the *FCGR3A* (rs396991; A>C)) and *FCGR2A* (rs1801274; A>G) polymorphisms with treatment response [84]. 

#### 2.5.2. CD52 Molecule; CD52

The *CD52* gene is located in chromosomal region 1p36.11 [85]. The Campath-1 family are monoclonal antibodies which recognize the CD52 antigen expressed in T and B lymphocytes, monocytes, natural killer cells, and macrophages. Through *CD52*, alemtuzumab gives rise to ADCC and CDC-mediated lysis after binding to the surface of T and B lymphocytes.

Although no studies have been described relating the influence of genetic alterations in the CD52 gene to the effectiveness of alemtuzumab in patients with MS, an association was observed in 108 Caucasian kidney transplant patients in Poland, where the rs1071849 (A>G; Asn40Ser) and rs17645 (A>G; Ile41Met) polymorphisms may have affected alemtuzumab response by altering the effectiveness of recognition of the C-terminal part of *CD52* [86].

### 2.6. Cladribine

**Figure 6:**
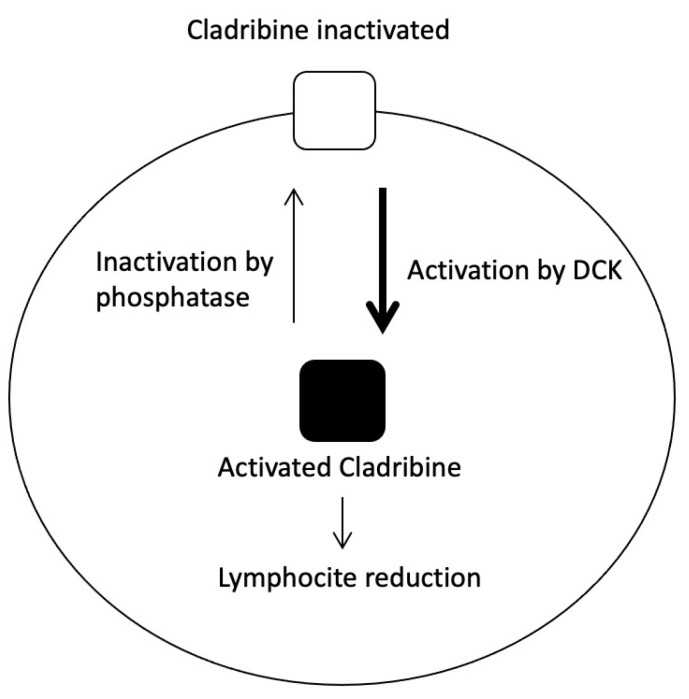
Mechanism of action of cladribine; DCK: Deoxycitidine Kinase.

Cladribine (Mavenclad^®^) is a deoxyadenosine nucleoside analogue prodrug. A substitution by chlorine on the purine ring protects cladribine from degradation by adenosine deaminase, which activates intracellular phosphorylation in lymphocytes [87]. Its active metabolite, cladribine triphosphate, accumulates in cells, giving rise to DNA damage and therefore to apoptosis [88]. The mechanism by which cladribine exerts its therapeutic effects in MS has not been fully clarified, but it is believed to produce a reduction in the number of B and T lymphocytes, interrupting the cascade of central immune events of MS (Figure 6) [89]. Cladribine reduces the annual attack rate by 58% [23]. However, there are alterations in genes implicated in cladribine’s mechanism of action that may lead to interindividual variability in response to the drug. 

#### 2.6.1. Ribonucleotide Reductase, M1 and M2 Subunits; RRM1 and RRM2

Ribonucleotide reductase (RR) is a key enzyme in the biosynthesis of deoxynucleotides, consisting of two subunits, M1 and M2 [90]. The genes that encode the two subunits are located on chromosomes 11p15.4 (*RRM1*) and 2p25.1 (*RRM2*) [91,92]. Greater RR activity or overexpression is one of the causes of resistance to cladribine, as it increases deoxynucleotide levels, which compete with cladribine nucleotides [93].

We have found no studies relating the presence of genetic alterations in these genes to drug response in MS patients, but an association has been observed in other demyelinating pathologies such as myeloid leukemia. A study of 90 patients of European ancestry in Utah (United States) and 90 of African ancestry in Nigeria with this disease observed that the *RRM1* promoter SNPs rs11030918 (C>T) (*p* = 0.04), rs12806698 (A>C) (*p* = 0.0004), and rs1042927 (A>C) (*p* = 0.030), and the *RRM2* SNP rs1138729 (A>C) (*p* = 0.001), were associated with higher mRNA expression and sensitivity to cytarabine [94].

#### 2.6.2. Adenosine Deaminase; ADA

The *ADA* gene encodes adenosine deaminase, an enzyme that catalyzes the irreversible deamination of adenosine and deoxyadenosine in the purine catabolic pathway. *ADA* is located on chromosome 20q13.12 [95]. The activity and the level of *ADA* have been reported to be altered in patients with MS compared to healthy subjects [96,97]. *ADA* is associated with the toxic accumulation of triphosphorylated deoxyadenosine and lymphocyte depletion, as shown in *ADA* genetic deficiency and in patients treated with cladribine [98].

A study of 561 Caucasian RRMS patients treated with cladribine showed that the allele C for the SNP rs244072 (T>C) was associated to higher expanded disability status scale (EDSS) (TT patients median = 1.5, Interquartile range (IQR) = 1–2.5; CT/CC median = 2; IQR = 1–3; *p* = 0.011) [99].

### 2.7. Siponimod

**Figure 7:**
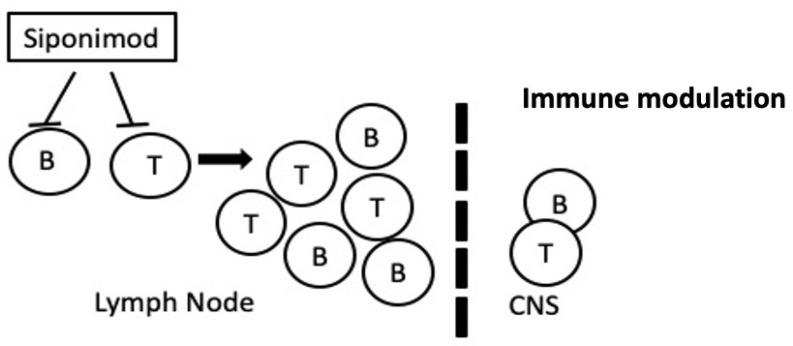
Mechanism of action of siponimod.

Siponimod (Mayzent^®^) is indicated for the treatment of RRMS patients with active disease defined by attacks or by imaging features of inflammatory activity. It is a sphingosine 1-phosphate (S1P) receptor modulator, which binds selectively to two of the five G-protein-coupled receptors for S1P (S1P1 and S1P5) [100]. By acting as a functional antagonist in lymphocyte S1P1 receptors, siponimod prevents the egress of lymphocytes from the lymph nodes, and therefore reduces the recirculation of T cells in the CNS and limits central inflammation (Figure 7) [101]. Siponimod reduces disability progression by 31% and the annual attack rate by 46% [19]. Although good results have been observed in response to the drug, there are various genetic factors that could affect interindividual variability. Although no studies evaluating the impact of pharmacogenetics on the effectiveness of this drug have been found, there are studies that describe the influence of pharmacogenetics on its safety. Siponimod is metabolized in the liver through the P450 cytochrome system (mainly through CYP2C9, subsequently through CYP3A4). The former, CYP2C9, is a polymorphic enzyme and genetic alterations in the *CYP2C9* gene could lead to greater exposure to siponimod and consequently greater toxicity [102,103]. In particular, renal clearance is higher in patients carrying the CYP2C9*1/*1 genotype (49.07 ± 3.04 μL/min/pmol) or the CYP2C9*2/*2 genotype (16.93 ± 0.29 μL/min/pmol) and lower in those with the CYP2C9*3/*3 genotype (4.37 ± 1.14 μL/min/pmol) [103]. Consequently, CYP2C9*3 heterozygotes should receive a lower dose and the CYP2C9*3/*3 genotype is a counterindication for siponimod.

### 2.8. Ocrelizumab

**Figure 8:**
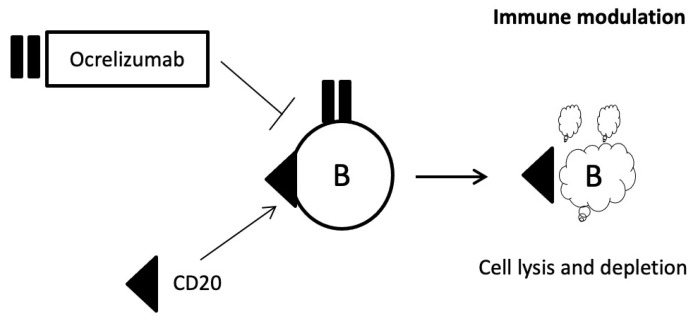
Mechanism of action of ocrelizumab.

Ocrelizumab (Ocrevus^®^) produces elimination of B lymphocytes by various mechanisms after binding to the CD20 glycoprotein, which is expressed on the cell membrane of B lymphocytes [104]. It is used to treat PPMS and is also indicated for RRMS (Figure 8) [105,106].

The drug reduces the appearance of attacks by 46% and the risk of disability progression by 40% [25]. Pharmacogenetics may play a crucial role in this variability in response to ocrelizumab. However, there are currently no publications relating genetics to ocrelizumab response in MS treatment.

## 3. Conclusions

This review has described genetic polymorphisms affecting the response to treatment with new MS therapies. However, certain limitations have been found in the existing literature in this area. Firstly, although some studies have evaluated the effects of SNPs in genes that encode proteins involved in the morbidities, metabolism, adverse effects, drug response, or mechanism of action of new therapies, their samples are small, making it difficult to generalize the role of these genes in new therapies. Studies with larger sample sizes are therefore required to confirm these results. Secondly, multiple co-existing SNPs could have a cumulative effect on the response to treatment with new therapies in relation to the mechanism of action, but there are almost no studies that analyze the concurrence of several polymorphisms in different genes. Finally, the existing studies show a lack of uniformity in their definitions of treatment response and too short a follow-up to generalize the results. In conclusion, further studies are needed, evaluating multiple SNPs in MS patients, with uniform treatment response criteria, longer-term follow-up, and larger sample sizes.

Multiple sclerosis is a disease with unpredictable development which can become disabling. The influence of polymorphisms in genes implicated in the pathologic environment, pharmacodynamics, and metabolism of the disease, or in the mechanism of action of the new therapies, on the effectiveness of these treatments has been previously investigated. Polymorphisms in the *GSTP1* (rs1695), *ITGA4* (rs1143676), *NQO1* (rs1800566), *AKT1* (rs2498804), and *GP6* (rs2304166) genes have shown an association with response to natalizumab, *ZMIZ1* (rs1782645) to fingolimod and dimethyl fumarate, *ADA* (rs244072) to cladribine, and *NOX3* (rs6919626) to dimethyl fumarate in patients with MS. Other genetic polymorphisms in other genes have been associated with the effectiveness of teriflunomide (*DHODH* (rs3213422)), alemtuzumab (*CD52* (rs107184 and rs17645)), and cladribine (*RRM1* (rs11030918 and rs12806698), and *RRM2* (rs1042927 and rs1138729)) in pathologies with features in common in MS. However, there are no studies evaluating the effect of pharmacogenetics on the effectiveness of other new therapies, such as siponimod and ocrelizumab.

In conclusion, the results suggest that the detection of polymorphisms in the *GSTP1*, *ITGA4*, *NQO1*, *AKT1*, *GP6*, *ZMIZ1*, *ADA*, and *NOX3* genes may be used in the future as a predictive marker of treatment response with new therapies in patients diagnosed with MS. However, more studies with larger sample sizes and longer follow-up are needed to confirm and extend the existing results.

## Figures and Tables

**Table 1:** Influence of gene polymorphisms on new therapies in multiple sclerosis.

Therapy	Gene	Population	N	Pathology	SNP	*p*-Value	OR (95% CI)	Genotype Associated	PMID
Dimethyl fumarate	*ZMIZ1*	Caucasian	39	MS	rs1782645	0.031	-	-	28063629
	*NOX3*	Caucasian	564	RRMS	rs6919626	0.036	1.57	G	31300673
Teriflunomide	*ABCG2*	Asian	24	Healthy Subjects	rs2231142	0.004	-	C	20972558
	*DHODH*	Caucasian	147	Rheumatoid Arthritis	rs3213422	0.048	1.98 (1.00–3.94)	C	19207032
Natalizumab	*GSTP1*	Caucasian	129	MS	rs1695	0.861	-	A	27993870
	*ITGA4*	Caucasian	117	MS	rs1143676	0.024	1.73 (1.07–2.81)	AG	25958306
	*NQO1*	Caucasian	124	MS	rs1800566	0.068		C	27993870
	*AKT1*	Caucasian	67	RRMS	rs2498804	<0.01	11.79 (3.72–37.38)	T	22577119
	*GP6*	Asian	67	MS	rs2304166	<0.01	22.18 (5.76–95.88)	C	20644114
Fingolimod	*ZMIZ1*	Caucasian	39	MS	rs1782645	0.039	-	-	29435643
Alemtuzumab	FCGR2A FCGR3A	Caucasian	85	RRMS	rs396991 rs1801274	>0.05	-	-	31682087
	CD52	Caucasian	108	Kidney transplant	rs107184 rs17645	-	-	-	20349607
Cladribine	*RRM1*	Caucasian African ancestry	90	Myeloid leukemia	rs11030918	0.04	-	-	24024897
					rs12806698	0.0004	-	-	
	*RRM2*				rs1042927	0.030	-	-	
					rs1138729	0.001	-	-	
	*ADA*	Caucasian	561	RRMS	rs244072	0.011	-	C	33007809
Siponimod	*CYP2C9*	-	-	MS	CYP2C9*1/*1 CYP2C9*2/*2 CYP2C9*3/*3	-	-	CYP2C9*3/*3	24024897
Ocrelizumab	-	-	-	-	-	-	-	-	-

## Data Availability

Not applicable.
